# Prokineticin-2 is associated with metabolic syndrome in a middle-aged and elderly Chinese population

**DOI:** 10.1186/s12944-015-0172-5

**Published:** 2016-01-05

**Authors:** Yong Wang, Xiaoyan Guo, Heng Ma, Lin Lu, Ruiyan Zhang

**Affiliations:** Department of Cardiology, Rui Jin Hospital, Shanghai Jiao Tong University School of Medicine, 197 Rui Jin 2nd Road, Shanghai, 200025 China; Department of Cardiology, Shanghai First People’s Hospital, Shanghai Jiao Tong University School of Medicine, Shanghai, 200080 China; Department of Gastroenterology, Gongli Hospital of Pudong New District, The Second Military Medical University, Shanghai, 200135 China; Yuhuangding Hospital, Qingdao University School of Medicine, Yantai, 264000 China; Institute of Cardiovascular Diseases, Shanghai Jiao Tong University School of Medicine, Shanghai, 200025 China

**Keywords:** Metabolic syndrome, Lipid disorders, Obesity, Diabetes mellitus, Prokineticin-2

## Abstract

**Background:**

Prokineticin-2 is confirmed to be involved in the inflammatory process. Inflammation plays an important role in the pathogenesis of metabolic syndrome (MS). However, whether prokineticin-2 is associated with MS or not remains unknown. Thus, we present this study to explore the association between prokineticin-2 and MS in a Chinese population.

**Methods:**

This study included 162 middle-aged and elderly Chinese patients with cardiovascular risk factors. The relationship between serum prokineticin-2 levels and various cardiometabolic risk factors, and MS were evaluated.

**Results:**

The participants with serum prokineticin-2 levels >6.32 ng/ml had increased waist circumference, body mass index (BMI), plasma triglyceride, diastolic blood pressure (DBP), blood glucose, and serum uric acid, but decreased age, plasma high-density lipoprotein cholesterol (HDL-C), and HDL-C/total cholesterol (TC) (all *P* < 0.05). A higher percentage of them had history of lipid disorders (19.3 vs 2.5 %, *P* = 0.001) and MS (77.1 vs 48.1 %, *P* < 0.001). Prokineticin-2 was positively correlated with TC (partial correlation coefficient: 0.233, *P* = 0.011), triglyceride (partial correlation coefficient: 0.504, *P <* 0.001), fasting plasma glucose (partial correlation coefficient: 0.336, *P* < 0.001), HbA1c (partial correlation coefficient: 0.285, *P* = 0.002), and uric acid (partial correlation coefficient: 0.234, *P* = 0.011) respectively, but was negatively correlated with HDL-C/TC (partial correlation coefficient: −0.269, *P* = 0.003) with adjustment for age, man, and BMI. Prokineticin-2 was significantly elevated in participants with MS (7.72 ± 3.34 *vs* 5.56 ± 2.39 ng/ml, *P* < 0.001). Furthermore, prokineticin-2 was significantly elevated in participants with increased numbers of MS components (5.17 ± 2.29 *vs* 5.94 ± 2.47 *vs* 7.13 ± 3.33 *vs* 8.32 ± 2.81 *vs* 9.82 ± 4.37 ng/ml*, P* for trend <0.001). Multiple logistic regression analysis indicated that prokineticin-2 was independently associated with MS (OR: 1.307, 95 % confidence interval: 1.127–1.515, *P* < 0.001) with adjustment for other potential confounders. If serum prokineticin-2 value can be considered as an indicator to discriminate MS, receiver operating characteristic curve analysis exhibited the area under the curve as 0.701.

**Conclusions:**

Prokineticin-2 is correlated with various cardiometabolic risk factors including blood lipid, blood glucose, blood pressure, BMI, and uric acid. And furthermore, the increased prokineticin-2 is independently associated with MS.

## Background

Metabolic syndrome (MS) is characterized as a cluster of metabolic abnormalities including obesity, dyslipidemia, hyperglycemia, and hypertension [1]. Patients with MS have significantly increased risks of the development of cardiovascular diseases [2]. Insulin resistance is the most common physiological and pathological mechanism shared by MS and these metabolic abnormalities [3]. Inflammation is also considered as the important mechanism of MS [4]. Various proinflammatory cytokines such as tumor necrosis factor-α (TNF-α), interleukin-6 (IL-6), and interleukin-1β (IL-1β) often participate in the pathogenesis of MS and insulin resistance [5, 6]. The proinflammatory state induces insulin resistance, leading to clinical and biochemical manifestations of the MS, such as obesity, lipid disorders, diabetes, and hypertension [7, 8].

Prokineticins are structural homologues of amphibian or reptilian peptide toxins which were first identified in the gastrointestinal tract [9], and have been isolated from bovine milk [10]. They comprise two classes: prokineticin-1 and prokineticin-2 (also called Bv8). Prokineticin-2 is confirmed to be involved in the inflammatory process. Prokineticin-2 is able to induce the macrophage to migrate and acquire a proinflammatory phenotype in mice. It can stimulate lipopolysaccharide-induced production of the proinflammatory cytokines IL-1β and interleukin-12 (IL-12), and reduce that of the anti-inflammatory cytokine interleukin-10 (IL-10) [11]. Prokineticin-2 is also highly expressed in inflamed murine tissues with infiltrating neutrophils and modulates inflammatory pain [12]. Another study has reported that prokineticin-2 can increase IL-1β, but decrease interleukin-4 (IL-4) and IL-10 production in mice splenocytes [13]. However, whether prokineticin-2 is associated with MS in which inflammation plays an important role is unknown to date. Thus, we present this study aimed to explore the association between serum prokineticin-2 levels and MS in a middle-aged and elderly Chinese population.

## Results

### Characteristics of study participants

Totally 162 middle-aged and elderly Chinese patients with cardiovascular risk factors were included in the final statistical analysis. Demographic and clinical features of these participants according to serum prokineticin-2 levels ≤6.32 or >6.32 ng/ml (median value) were shown in Table 1. The average age was 61.8 ± 11.1 years old (range from 42 to 88). 102 participants (63.0 %) were man. The serum prokineticin-2 levels were range from 2.30 to 16.08 ng/ml. The prevalence of MS in this population was 63.0 %.

**Table 1:** Clinical characteristics of patients with prokineticin-2 ≤ 6.32 or >6.32 ng/ml

Variables	All (*n* = 162)	Prokineticin-2 ≤ 6.32 (*n* = 79)	Prokineticin-2 > 6.32 (*n* = 83)	*P* value
Age (yrs)	61.8 ± 11.1	63.9 ± 11.6	59.7 ± 10.3	0.014
Men (n, %)	102(63.0 %)	44(55.7 %)	58(69.9 %)	0.062
Current smoking (n, %)	38(25.3 %)	18(24.3 %)	20(26.3 %)	0.779
Current drinking (n, %)	8(5.5 %)	4(5.6 %)	4(5.4 %)	0.968
Type 2 diabetes (n, %)	34(21.0 %)	12(15.2 %)	22(26.5 %)	0.077
Hypertension (n, %)	106(65.4 %)	50(63.3 %)	56(67.5 %)	0.576
History of lipid disorders (n, %)	18(11.1 %)	2(2.5 %)	16(19.3 %)	0.001
MS (n, %)	102(63.0 %)	38(48.1 %)	64(77.1 %)	<0.001
Waist circumference (cm)	89(79–96)	87(78–94)	90(80–97)	0.015
BMI (kg/m^2^)	25.7 ± 3.29	25.1 ± 3.48	26.3 ± 3.01	0.019
SBP (mmHg)	133(122–149)	132(121–151)	135(122–148)	0.536
DBP (mmHg)	77.0 ± 11.0	75.1 ± 11.2	78.8 ± 10.6	0.030
Triglyceride (mmol/l)	1.76 ± 1.63	1.29 ± 0.56	2.21 ± 2.13	<0.001
Total cholesterol (mmol/l)	4.25 ± 1.31	4.08 ± 1.20	4.41 ± 1.39	0.114
LDL-C (mmol/l)	2.47 ± 1.02	2.41 ± 1.02	2.53 ± 1.03	0.478
HDL-C (mmol/l)	1.06 ± 0.30	1.13 ± 0.32	0.98 ± 0.27	0.002
HDL-C/TC	0.27 ± 0.10	0.30 ± 0.11	0.24 ± 0.09	0.001
HbA1c (%)	6.18 ± 0.97	5.95 ± 0.59	6.35 ± 1.15	0.017
Fasting plasma glucose (mmol/l)	5.47 ± 1.48	5.18 ± 1.13	5.74 ± 1.71	0.015
2 h plasma glucose (mmol/l)	8.15 ± 3.07	7.39 ± 2.50	8.90 ± 3.40	0.004
Serum creatinine (umol/l)	81(70–90)	79(69–90)	81(70–91)	0.547
Uric acid (umol/l)	330 ± 77.2	306 ± 69.1	353 ± 77.7	<0.001
Lipid-lowering treatment (n, %)	110(68.8 %)	50(64.9 %)	60(72.3 %)	0.316
Anti-hypertension therapy (n, %)	86(53.1 %)	42(53.2 %)	44(53.0 %)	0.984
Hypoglycemic treatment (n, %)	20(12.5 %)	6(7.8 %)	14(16.9 %)	0.083

*MS* metabolic syndrome, *BMI* body mass index, *SBP* systolic blood pressure, *DBP* diastolic blood pressure, *LDL-C* low-density lipoprotein cholesterol, *HDL-C* high-density lipoprotein cholesterol, *HDL-C/TC* high-density lipoprotein cholesterol/total cholesterol, *HbA1c* glycated hemoglobin. Values are means ± SD, medians (interquartile range), or numbers with percentage in parenthesis

The participants with serum prokineticin-2 levels >6.32 ng/ml had increased waist circumference, body mass index (BMI), diastolic blood pressure (DBP), plasma triglyceride, glycated hemoglobin (HbA1c), fasting plasma glucose, 2 h plasma glucose, and serum uric acid levels, but decreased age, plasma high-density lipoprotein cholesterol (HDL-C) levels, and high-density lipoprotein cholesterol/total cholesterol (HDL-C/TC) ratios than those with prokineticin-2 ≤ 6.32 (all *P* < 0.05). A higher percentage of participants with serum prokineticin-2 levels >6.32 ng/ml had history of lipid disorders, and MS than those with prokineticin-2 ≤ 6.32 (both *P* < 0.01). The lipid-lowering treatment was not statistically different between two groups (*P* > 0.05). The difference in serum prokineticin-2 levels in patients with and without diabetes was not significant (7.65 ± 3.50 ng/ml, *n* = 34, *vs* 6.72 ± 3.09 ng/ml, *n* = 128, *P* = 0.131). The difference in serum prokineticin-2 levels in patients with and without hypertension was also not significant (7.00 ± 3.24 ng/ml, *n* = 106, *vs* 6.76 ± 3.12 ng/ml, *n* = 56, *P* = 0.662).

### Correlation between serum prokineticin-2 levels and cardiometabolic risk factors

The data in Table 1 implied that serum prokineticin-2 levels were possibly associated with some cardiometabolic risk factors. Thus, we used simple linear correlation to evaluate the correlation among them respectively. We found that serum prokineticin-2 levels were positively correlated with BMI, DBP, TC, triglyceride, fasting plasma glucose, 2 h plasma glucose, HbA1c, and uric acid levels respectively (all *P* < 0.05, Table 2), whereas were negatively correlated with age, HDL-C, and HDL-C/TC respectively (all *P* < 0.05, Table 2).

**Table 2:** Correlation between various factors and serum prokineticin-2 levels (ng/ml)

Variables	Correlation coefficient	*P* value	Partial correlation coefficient	*P* value
Age (yrs)	−0.284	<0.001	-	-
BMI (kg/m^2^)	0.245	0.002	-	-
SBP (mmHg)	0.019	0.806	0.076	0.414
DBP (mmHg)	0.199	0.011	0.126	0.176
Total cholesterol (mmol/l)	0.251	0.001	0.233	0.011
Triglyceride (mmol/l)	0.392	<0.001	0.504	<0.001
LDL-C (mmol/l)	0.133	0.096	0.099	0.289
HDL-C (mmol/l)	−0.202	0.011	−0.088	0.346
HDL-C/TC	−0.357	<0.001	−0.269	0.003
HbA1c (%)	0.331	<0.001	0.285	0.002
Fasting plasma glucose (mmol/l)	0.307	<0.001	0.336	<0.001
2 h plasma glucose (mmol/l)	0.253	0.004	0.167	0.071
Uric acid (umol/l)	0.342	<0.001	0.234	0.011

*BMI* body mass index, *SBP* systolic blood pressure, *DBP* diastolic blood pressure, *LDL-C* low-density lipoprotein cholesterol, *HDL-C* high-density lipoprotein cholesterol, *HDL-C/TC* high-density lipoprotein cholesterol/total cholesterol, *HbA1c* glycated hemoglobin. Correlation coefficient was calculated using simple linear correlation analysis without adjustment. Partial correlation coefficient was calculated using partial correlation analysis with adjustment for age, man, and BMI

Furthermore, correlation between serum prokineticin-2 levels and these factors was determined using partial correlation analysis with adjustment for age, man, and BMI. The data in Table 2 showed that prokineticin-2 were still positively correlated with TC, triglyceride, fasting plasma glucose, HbA1c, and uric acid levels respectively (all *P* < 0.05), but were negatively correlated with HDL/TC (*P* < 0.01).

### Association between serum prokineticin-2 levels and MS

**Fig. 1:**
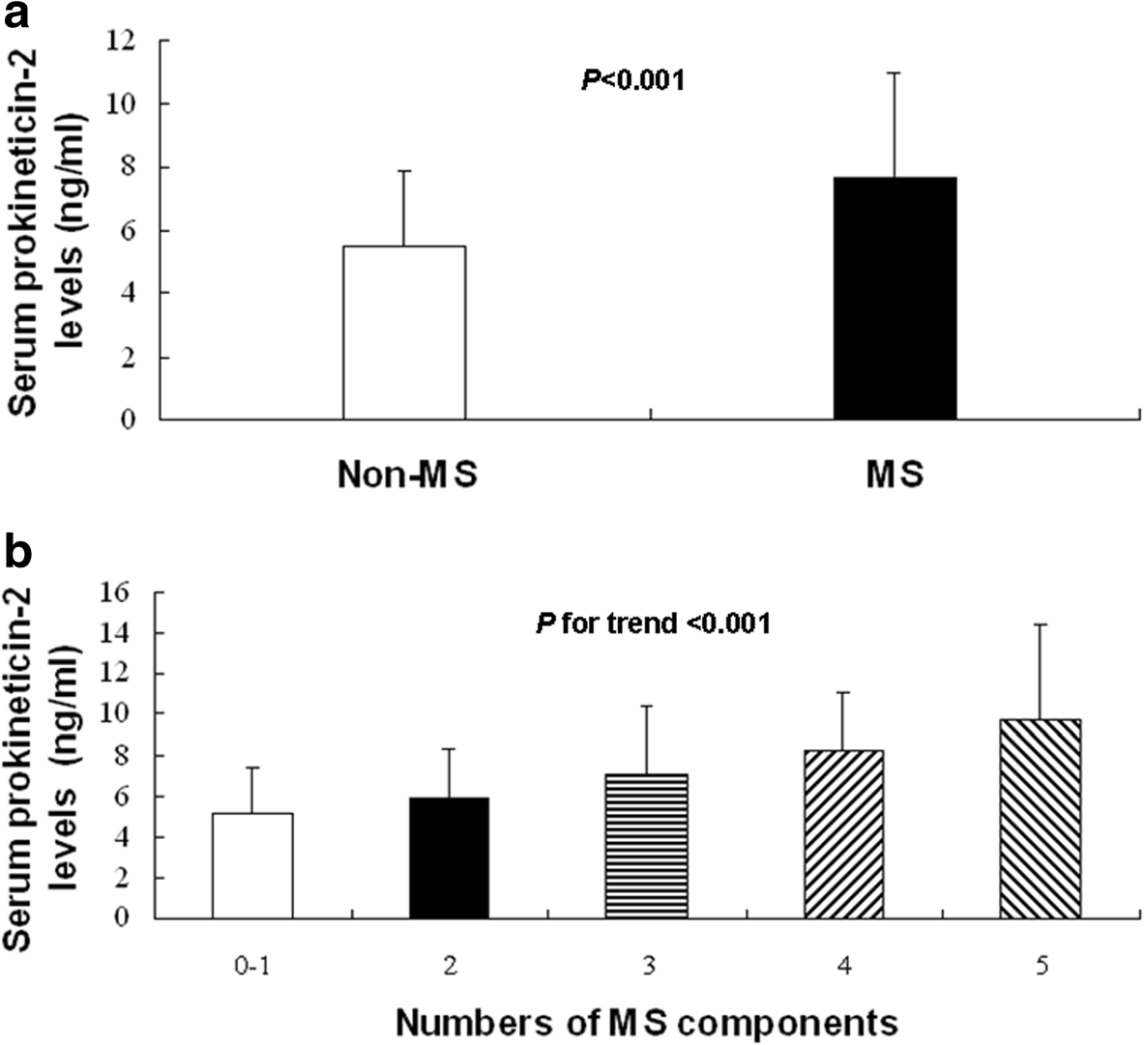
Association between MS and serum prokineticin-2 levels. **a**. Serum prokineticin-2 levels in patients with and without MS. Non-MS: 5.56 ± 2.39 ng/ml, *n* = 60; MS: 7.72 ± 3.34 ng/ml, *n* = 102, *P* < 0.001. **b**. Serum prokineticin-2 levels were elevated with increased numbers of MS components. 0–1: 5.17 ± 2.29 ng/ml, *n* = 30; 2: 5.94 ± 2.47 ng/ml, *n* = 30; 3: 7.13 ± 3.33 ng/ml, *n* = 62; 4: 8.32 ± 2.81 ng/ml, *n* = 32; 5: 9.82 ± 4.37 ng/ml, *n* = 8, *P* for trend <0.001

Most of the cardiometabolic risk factors in the context were important components of MS. Thus, we attempted to explore characteristics of the serum prokineticin-2 levels in participants with MS and MS components. Figure 1 showed that serum prokineticin-2 levels were significantly elevated in participants with MS than those without MS (7.72 ± 3.34 *vs* 5.56 ± 2.39 ng/ml, *P* < 0.001). Furthermore, serum prokineticin-2 levels were significantly elevated in participants with increased numbers of MS components (5.17 ± 2.29 *vs* 5.94 ± 2.47 *vs* 7.13 ± 3.33 *vs* 8.32 ± 2.81 *vs* 9.82 ± 4.37 ng/ml*, P* for trend <0.001, Fig. 1).

In order to explore the independent association between serum prokineticin-2 levels and MS, multiple stepwise logistic regression analysis was performed with adjustment for other potential confounders. Table 3 indicated that odds ratio (OR) of serum prokineticin-2 levels to MS was 1.294 (95 % confidence interval: 1.139–1.470, *P* < 0.001) with adjustment for age and men in model 1. Furthermore, OR of prokineticin-2 to MS was 1.240 (95 % confidence interval: 1.084–1.419, *P* = 0.002) with adjustment for age, men, and BMI in model 2, or 1.307 (95 % confidence interval: 1.127–1.515, *P* < 0.001) with adjustment for age, men, BMI, hypertension, history of lipid disorders, type 2 diabetes, lipid-lowering treatment, anti-hypertension therapy, and hypoglycemic treatment in model 3 respectively.

**Table 3:** Independence of serum prokineticin-2 levels (ng/ml) associated with MS

Groups	OR	95 % confidence interval	*P* value
Model 1	1.294	1.139–1.470	<0.001
Model 2	1.240	1.084–1.419	0.002
Model 3	1.307	1.127–1.515	<0.001

Multiple stepwise logistic regression analysis was used to calculate the odds ratio (OR) of serum prokineticin-2 levels associated with MS with adjustment for other potential confounders. Model 1: Adjustment for age and men; Model 2: Adjustment for age, men, and BMI; Model 3: Adjustment for age, men, BMI, hypertension, history of lipid disorders, type 2 diabetes, lipid-lowering treatment, anti-hypertension therapy, and hypoglycemic treatment

### Discriminating power of prokineticin-2 to detect MS

**Fig. 2:**
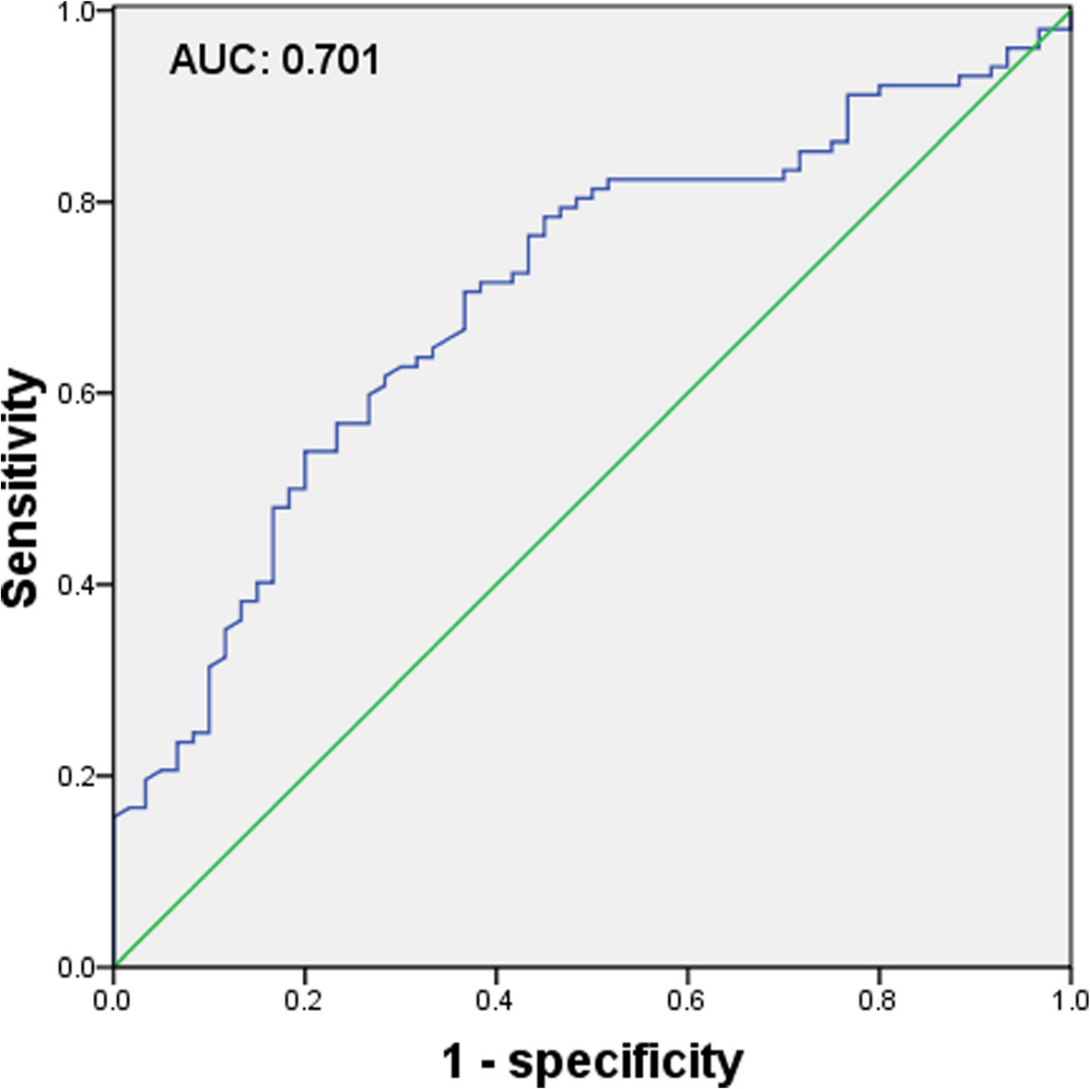
The discriminating power of prokineticin-2 to detect MS. ROC curve was used to evaluate the discriminating power of serum prokineticin-2 levels to detect MS. Area under the curve (AUC) was 0.701

If serum prokineticin-2 values can be considered as an indicator to discriminate MS, we would use receiver operating characteristic (ROC) curve analysis to assess the discriminating power of serum prokineticin-2 levels to detect MS. The ROC curve was displayed in Fig. 2. Area under the curve (AUC) was 0.701 (95 % CI: 0.620–0.783, *P <* 0.001). If a serum prokineticin-2 level equal to 5.72 was considered as a cut off value, the sensitivity to discriminate MS would be 0.716, and the specificity would be 0.617.

### Post-hoc sample size calculation

After we finished analyzing the data and detected the differences in serum prokineticin-2 levels between subjects with and without MS, we performed a post-hoc sample size calculation in order to test if the sample size in this study was sufficient. We set the type I error (α) as 0.05, type II error (β) as 0.1, and the power (1-β) as 0.9. Also, the differences in mean of serum prokineticin-2 levels between two groups and standard deviation were included in the sample size calculation. Then the results suggested that the sample size should not be less than 40. Thus, the sample size (*n* = 162) in this study was sufficient.

## Discussion

MS is highly associated with increased morbidity and mortality of cardiovascular diseases, which are placing an increasing burden on healthcare resources in China. Insulin resistance and inflammation are considered as the pivotal mechanisms involved in the pathogenesis of MS. However, in recent years there is still increasing attention focusing on the pathophysiologic mechanisms of MS. Lots of adipokines and proinflammatory mediators such as leptin, adiponectin, chemerin, TNF-α, IL-1, IL-6, retinol-binding protein-4, and serum amyloid-A are found to be associated with the development of MS [14–16]. In this study, we have explored the clinical features of patients according to prokineticin-2 and found it a novel and potential protein associated with MS.

The data in this study displayed that participants with elevated prokineticin-2 had a higher percentage of history of lipid disorders. They also had elevated waist circumference, BMI, DBP, plasma triglyceride, blood glucose, and serum uric acid, but reduced HDL-C and HDL-C/TC. To be noted, the changes in these metabolic profiles are often detected in patients with MS. The further correlation between prokineticin-2 and BMI, blood lipid profiles, blood glucose, and serum uric acid with adjustment for potential confounders confirmed that prokineticin-2 is strongly associated with the components of MS.

We have reviewed the literatures on prokineticin-2 and have not found studies on whether prokineticin-2 is associated with blood lipid profiles, blood pressure, or serum uric acid. A previous study exhibited that peripheral administration of recombinant prokineticin-2 protein can reduce food intake and body weight in mice [17]. The authors in that research also reported that intraperitoneal injection of prokineticin-2 protein did not significantly affect blood glucose. However, the serum levels of prokineticin-2 in human associated with body weight and blood glucose has not been investigated to date. In our study, serum prokineticin-2 levels were elevated accompanied by increased BMI in patients. Nevertheless, we have not known yet the mechanism of the elevation in serum prokineticin-2 levels in human with higher body weight in this study. Thus, the direct roles of prokineticin-2 in metabolic profiles still need further detailed studies in the future. In fact, abnormalities in cardiometabolic risk factors including lipid disorders are important cause for coronary heart disease and mortality. Efficient control of blood lipid can lead to a reduction in cardiovascular risk. Lots of therapy such as statins and various nutraceuticals were found able to improve lipid profiles and reduce cardiovascular events [18]. The new finding in this study that prokineticin-2 is strongly associated with cardiometabolic risk factors including lipid disorders might help to offer better understanding of cardiometabolic disorders and cardiovascular diseases, and further improve the therapy in the future possibly.

There was a high prevalence of MS (63.0 %) in this population because the enrolled study subjects were relatively older, and had more cardiovascular risk factors. This study indicated that a higher percentage of participants with elevated prokineticin-2 were diagnosed with MS. At the same time, patients with MS had higher prokineticin-2. The data also showed that serum levels of prokineticin-2 in patients with all five MS components were almost doubled compared with those without or with only one MS component. And furthermore, prokineticin-2 was independently associated with MS with adjustment for other potential confounders. ROC curve analysis also suggested that the discriminating power of prokineticin-2 to detect MS was moderate, and further confirmed the association between them. There are no literatures which have described the relationship between prokineticin-2 and MS to date. Thus, we think that we could possibly supply some new data on MS from this study.

Nevertheless, what is the causal relationship between prokineticin-2 and MS? What is the role of the elevated prokineticin-2 levels in blood lipid profiles, blood glucose, blood pressure, and pathogenesis of MS? It is also unclear if the relationship between prokineticin-2 and metabolic syndrome is primary or secondary to other proinflammatory cytokines and/or adipokines. These topics are not explored and are the limitations of this study. The potential mechanism remains to be elucidated and need further studies. There is no healthy control group. This is also a limitation of this study.

## Conclusions

In summary, prokineticin-2 is correlated with many cardiometabolic risk factors including blood lipid, blood glucose, blood pressure, BMI, and uric acid. And furthermore, the increased prokineticin-2 is independently associated with MS. Thus, the data in this study might be helpful to a better understanding of the pathophysiological alterations of MS.

## Methods

### Study subjects

This study included middle-aged and elderly Chinese patients with cardiovascular risk factors. The enrolled participants (*n* = 162) were over 40 years old and hospitalized in department of cardiology in Shanghai Rui Jin hospital from May to July 2015. Cardiovascular risk factors included elder age, smoking, overweight or obesity, family history of cardiovascular diseases, hypertension, type 2 diabetes, or history of lipid disorders. Exclusion criteria included acute infected diseases, severe liver failure, uremia, pregnancy, mental disorder, or cancer. This study complied with the Declaration of Helsinki. It was also approved by the ethics committee of Shanghai Jiao Tong University. Informed consent was obtained from all the participants prior to enrollment in the study.

### Serum prokineticin-2 levels examination

Peripheral blood from each subject was centrifuged at 3,000 rpm for 20 min. Serum from supernatant was collected and immediately stored at −80 °C until being used. Serum prokineticin-2 levels were assayed using competitive enzyme linked immunosorbent assay kits (LifeSpan BioSciences, Inc., Seattle, WA) according to the manufacturer’s instructions. The assay had excellent specificity to recombinant and natural human prokineticin-2. No significant cross-reactivity or interference between between prokineticin-2 and analogs was observed. The intra-assay and inter-assay precision of this kit was evaluated. 3 samples with low, middle, and high level prokineticin-2 were tested 20 times on one plate respectively for evaluating intra-assay precision. 3 samples with low, middle, and high level prokineticin-2 were tested on 3 different plates, 8 replicates in each plate for evaluating inter-assay precision. CV was calculated as standard deviation/mean. The intra-assay CV was less than 10 %, and inter-assay CV was less than 12 %. The minimum detectable concentration of each kit was 0.093 ng/ml for prokineticin-2. Duplicate assay was performed for each serum sample and the results were expressed as ng/ml. The results of assay were generated by one personnel blinded to the clinical characteristics of the study participants.

### Metabolic syndrome definition

MS was defined according to the National Cholesterol Education Program Adult Treatment Panel III criteria with modification [19–21]. A subject was diagnosed with MS when three or more of the following were satisfied: (1) Overweight or obesity: waist circumference >90 cm in men, >80 cm in women, or BMI ≥25 kg/m^2^ in both sexes; (2) Hypertriglyceridaemia: triglyceride ≥1.7 mmol/l or the use of lipid lowering agents due to elevated triglyceride; (3) Low HDL-C: HDL-C < 1.03 mmol/l in men and <1.29 mmol/l in women; (4) Elevated blood pressure: blood pressure ≥130/85 mmHg or taking antihypertensive drugs due to hypertension; (5) Elevated blood glucose: fasting plasma glucose ≥5.6 mmol/l or previously diagnosed type 2 diabetes.

### Clinical data collection

A case report form was developed to assess the general characteristics, clinical diagnosis, medical treatment, and biochemical examination. BMI was calculated as body weight in kilograms divided by body height in meters squared (kg/m^2^). Waist circumference was measured at the middle point between the costal margin and iliac crest. Current smoking was determined when subjects were smoking currently and more than one cigarette daily in at least one year continuously. The definition of drinking was currently drinking liquor, beer or wine at least for a year. Overweight was confirmed as a BMI of 25 to less than 30 and obesity as a BMI of 30 or higher. History of lipid disorders meant that total cholesterol was ≥ 5.7 mmol/l, or low-density lipoprotein cholesterol (LDL-C) was ≥3.6 mmol/l, or triglyceride ≥1.7 mmol/l, or treatment with antihyperlipidemic agents due to hyperlipidemia. Hypertension was diagnosed when systolic blood pressure (SBP) ≥140 mmHg, or DBP ≥90 mmHg, or being actively treated with anti-hypertension drugs. Type 2 diabetes was diagnosed by a fasting plasma glucose test showing ≥7.0 mmol/l, or by a random plasma glucose test showing ≥11.1 mmol/l, or when they were actively receiving therapy using insulin or oral medications for diabetes, and with the exclusion of type 1 diabetes. Lipid-lowering treatment included the use of statins or other lipid-lowering agents.

### Statistical analysis

Data were analyzed using the software program SPSS 13.0 (SPSS Inc., Chicago, IL, USA). The continuous variables with normal distribution were expressed as the mean ± standard deviation, whereas variables with a skewed distribution were reported as the median (interquartile range). Categorical variables were expressed as frequency and percentage. The chi-square test was used to compare categorical variables between several groups. The independent-sample *t*-test or Mann–Whitney *U* test were used to compare continuous variables with normal or skewed distribution between two groups respectively. Trend test were used to compare continuous variables among more than two groups respectively. Relationship between two variables was tested using simple linear correlation and partial correlation analysis respectively. Multiple stepwise logistic regression analysis was used to assess the independence of the association between serum prokineticin-2 levels and MS with adjustment of other potential confounders. The OR and 95 % confidence interval were calculated. ROC curve analysis was used to assess the discriminating power of serum prokineticin-2 levels to detect MS. P < 0.05, which is two-sided, was considered significant.

## Abbreviations

MS: Metabolic syndrome
TNF-α: Tumor necrosis factor-α
IL-6: Interleukin-6
IL-1β: Interleukin-1β
IL-12: Interleukin-12
IL-10: Interleukin-10
IL-4: Interleukin-4
BMI: Body mass index
SBP: Systolic blood pressure
DBP: Diastolic blood pressure
HbA1c: Glycated hemoglobin
LDL-C: Low-density lipoprotein cholesterol
HDL-C: High-density lipoprotein cholesterol
TC: Total cholesterol
HDL-C/TC: High-density lipoprotein cholesterol/total cholesterol
OR: Odds ratio
ROC: Receiver operating characteristic
AUC: Area under the curve
